# Switching the N-Capping Region from all-L to all-D Amino Acids in a VEGF Mimetic Helical Peptide

**DOI:** 10.3390/molecules27206982

**Published:** 2022-10-17

**Authors:** Lucia De Rosa, Donatella Diana, Domenica Capasso, Rachele Stefania, Rossella Di Stasi, Roberto Fattorusso, Luca Domenico D’Andrea

**Affiliations:** 1 Istituto di Biostrutture e Bioimmagini, Consiglio Nazionale delle Ricerche, Via Pietro Castellino 111, 80131 Napoli, Italy; 2 CESTEV, Università di Napoli “Federico II”, Via de Amicis 95, 80134, Napoli, Italy; 3 CIRPEB, Università di Napoli “Federico II”, Via Mezzocannone 8, 80134 Napoli, Italy; 4 Dipartimento di Scienze e Innovazione Tecnologica, Università del Piemonte Orientale “Amedeo Avogadro”, Viale Teresa Michel 11, 15120 Alessandria, Italy; 5 Dipartimento di Scienze e Tecnologie Ambientali, Biologiche e Farmaceutiche, Università della Campania “Luigi Vanvitelli”, Via Vivaldi 43, 81100 Caserta, Italy; 6 Istituto di Scienze e Tecnologie Chimiche “Giulio Natta”, Consiglio Nazionale delle Ricerche, Via M. Bianco 9, 20131 Milano, Italy

**Keywords:** peptide folding, α-helix, N-capping, D-amino acid, NMR, peptide conformation

## Abstract

The N-capping region of an α-helix is a short N-terminal amino acid stretch that contributes to nucleate and stabilize the helical structure. In the VEGF mimetic helical peptide QK, the N-capping region was previously demonstrated to be a key factor of QK helical folding. In this paper, we explored the effect of the chiral inversion of the N-capping sequence on QK folding, performing conformational analysis in solution by circular dichroism and NMR spectroscopy. The effect of such a modification on QK stability in serum and the proliferative effect were also evaluated.

## 1. Introduction

The α-helix is the most abundant secondary structure element found in protein structure. It has been extensively studied and the molecular factors governing its folding and stability have been deeply analyzed [1,2,3,4,5,6]. As a consequence, helical peptides can now be routinely designed for disparate applications. In particular, they are advantageous tools to modulate protein–protein interactions. The main factors determining peptide helix folding and stability have been described in depth: amino acid propensity, capping regions, intra-helix interactions, acetylation/amidation of terminal ends, and covalent constrains. The N-capping motif is an amino acid segment preceding the start of an α-helix, composed of amino acids with backbone dihedral angles not assuming the characteristic α-helix values. This region stabilizes α-helix conformation through additional hydrogen bonds to the uncapped amide protons of the first helical turn and specific hydrophobic interaction between side chains [7]. These structural elements have been observed in protein region preceding the α-helix sequence [8]. The N-capping region can be designed to stabilize short helical peptides, which, without a specific scaffold, usually show a low to middle helical content and frayed ends in aqueous solution.

We reported in 2005 a de novo designed VEGF mimetic short helical peptide (QK) that has been deeply characterized, shedding light on its biological [9,10,11,12,13] and structural [14,15,16,17,18] properties. Peptide QK is a 15-mer VEGF receptors binder peptide [19] with proangiogenic activity [20,21] belonging to a set of designed conformationally constrained peptides [22,23,24,25,26,27,28] mimicking VEGF secondary structure elements involved in receptor interaction. It is composed only of natural amino acid and was designed to reproduce the VEGF α1 helix (VEGF residues 17–25) [29]. Outside the helical segment, N- and C-capping regions were appended [9]. The peptide assumes in solution a well-defined α-helical conformation spanning the peptide segment 4–12, while the terminal ends are mainly frayed as expected for short peptide in solution [9]. The C-terminal region is not playing a role in peptide stability and biological activity [16]. Instead, N-terminal capping residues (Lys-Leu-Thr) are essential for peptide folding and biological activity. In fact, the deletion of this peptide segment resulted in an unfolded and biologically inactive peptide [15]. However, amino acid deletion entails a substantial modification of peptide physical properties, such as amino acid composition, length, net charge, and global hydrophobicity, which could drastically impact on peptide folding propensity and biological effect. We reasoned that the chiral inversion of the N-Cap could effectively abolish the contribution of the N-cap to helical stability without altering the peptide physical properties. In this paper, we replaced the N-terminal amino acids Lys-Leu-Tyr of QK with the corresponding D-enantiomers, ^D^Lys-^D^Leu-^D^Thr, and we evaluated the effect of such a modification on peptide folding, function, and serum stability.

## 2. Results

Peptide D-QK (Ac-^d^Lys-^d^Leu-^d^Thr-Trp-Gln-Glu-Leu-Tyr-Gln-Leu-Lys-Tyr-Lys-Gly-Ile-NH_2_) was synthesized by standard Fmoc chemistry on solid phase. It was obtained in good yield and with a purity >95% (Figure S1). 

### 2.1. Circular Dichroism Analysis

**Figure 1:**
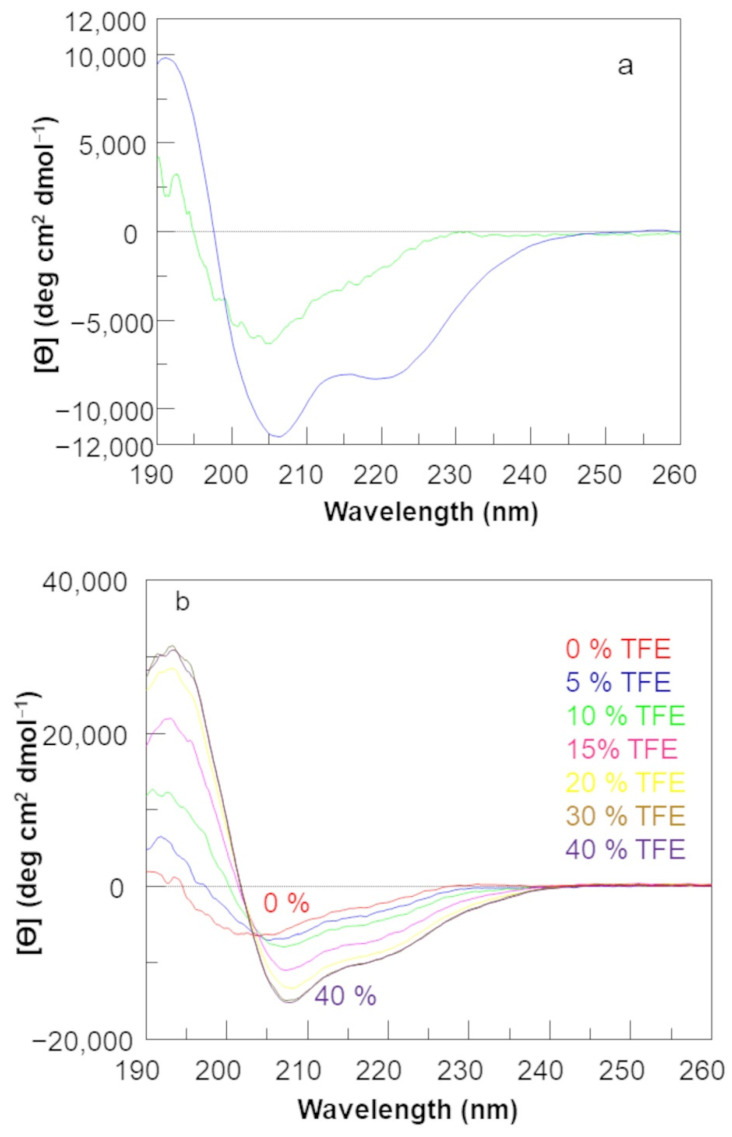
(**a**) Far-UV circular dichroism spectrum of D−QK (green line) and QK (blue line) recorded at 20 °C in 5 mM phosphate buffer, pH 7.0. (**b**) Trifluoroethanol titration of peptide D-QK. CD spectra were recorded at 20 °C in water/TFE mixture. TFE percentage was increased from 0% to 40%. Spectra are reported as mean residue ellipticity.

The conformational analysis in solution by far-UV circular dichroism (CD) of the D-QK peptide was performed in phosphate buffer at pH 7.0 and at 20 °C (Figure 1). For comparison, the CD spectrum of QK peptide was also recorded in the same experimental conditions (Figure 1).

The CD spectrum of QK shows all the spectral characteristics of the α-helical structure (two well defined minima around 208 and 222 nm, a crossover point at 200 nm, and a maximum around 190 nm), while the CD spectrum of D-QK has these features less remarkable. The minimum at 208 nm appears slightly blue-shifted, while the minimum at 222 nm is indistinguishable. Besides, both the negative band around 208 nm and the positive band around 190 nm are less pronounced with respect to the QK CD spectrum. The data suggest that the D-QK assumes in solution a less ordered α-helical conformation. The helical content of peptide D-QK is about five times less than QK as estimated from the ellipticity value at 222 nm [30]. In order to verify the propensity of D-QK to assume a helical conformation, we performed a titration experiment with trifluoroethanol (TFE), an organic cosolvent able to stabilize the helical conformation in peptide molecules [31]. The Far-UV CD spectrum of D-QK was recorded in a water/TFE mixture (Figure 1b) with the TFE percentage progressively increased from 0% to 40% [32]. CD spectra of D-QK in presence of TFE are suggestive of a transition towards a better-defined helical conformation. By increasing the percentage of TFE, the first minimum in the CD spectrum of D-QK progressively shifts towards 208 nm, becoming centered at this wavelength at 20% of TFE. Besides, the minimum at 222 nm became progressively more pronounced. An isodichroic point is clearly present at 203 nm. At 40% TFE, the helical content is increased about five times with respect to the peptide in water without TFE.

### 2.2. Structural Investigation by NMR Spectroscopy

**Figure 2:**
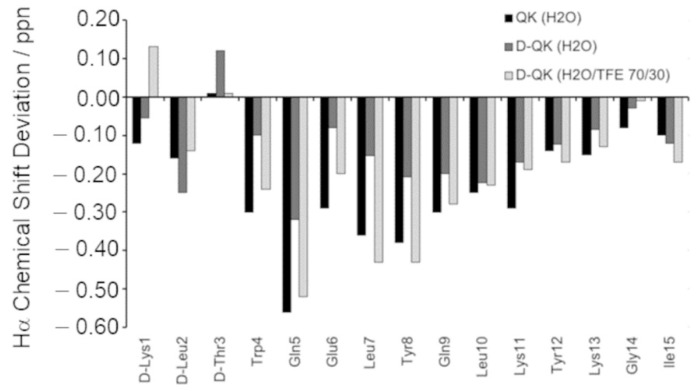
Experimental secondary chemical shifts from the Hα protons of QK (in H_2_O), D-QK (in H_2_O) and D-QK (H_2_O/TFE 70/30 *v/v*).

The conformational properties of D-QK peptide were first investigated in H_2_O at pH 5.5, under the same conditions used previously for QK [9]. To this aim, different 2D [1H, 1H] NMR spectra were recorded, and the analysis of TOCSY and NOESY experiments allowed to obtain complete peptide proton resonance assignments (Table S1). Meanwhile, the analysis of DQF-COSY experiment allowed to obtain the coupling constants (Table S2). The analysis of chemical shifts for Hα protons with respect to the random coil reference values (chemical shift deviations, CSDs), calculated according to Wishart [33] indicates a negative trend and thus the possible presence of an alpha-helix in the fragment encompassing residues from Trp4 to Tyr12 (Figure 2). However, many CSD values of QK appear lower than those of QK (Figure 2), with a percentage of helical population, estimated from CSD values, equal to 30% [34]. Particularly, when comparing the D-QK CSD values with those of QK, the main, albeit very small, differences concern the region encompassing residues from Trp4 to Tyr8. These data might be ascribed to the effect of the D-amino acids on the helical propensity of their neighbors.

**Figure 3:**
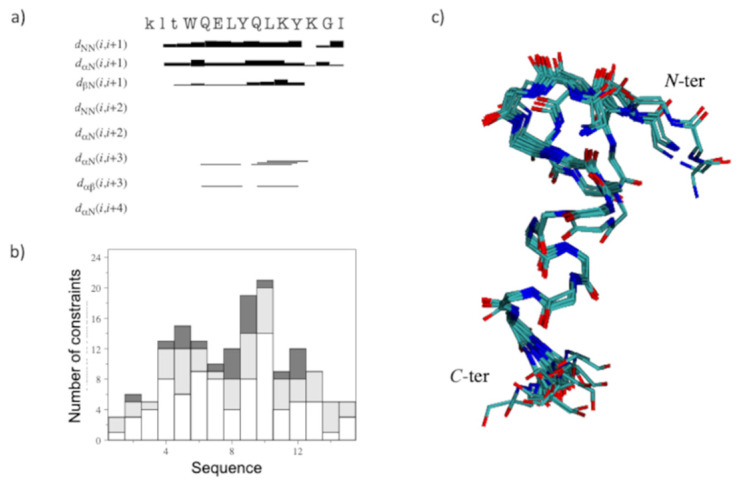
(**a**) D-QK NOEs diagram in water. The amino acid sequence is reported on the top. Principal sequential and medium-range NOEs are shown; the thickness of each block is proportional to the corresponding NOE intensity. (**b**) Number of constraints per residue. White, light gray and dark gray bars indicate intra-residue, sequential, and medium-range connectivities, respectively. (**c**) Superposition of the minimized 20 best structures represented as neon (RMSD (N, Ca, C’(5–12) = 0.37 ± 0.05 Å). N-ter and C-ter refer to the amino and carboxyl termini, respectively.

The NOESY assignments showed a reduced number of NOE cross-peaks than those to QK, which are mostly localized in the region of D-QK encompassing residues Gln9-Tyr12. Furthermore, the presence of dNN (i, I + 1) and dαN (i, I + 3) distance constraints together with the CSD analysis suggested the existence of a residual helical structure in this region of the peptide (Figure 3a).

**Figure 4:**
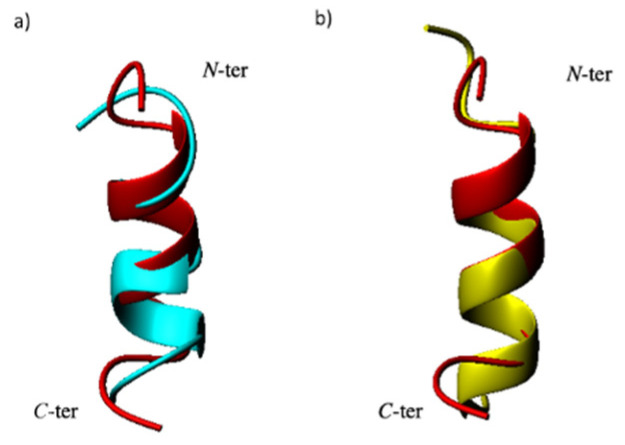
(**a**) Superposition of QK (red) and representative structure of D-QK (cyan) in aqueous solution (RMSD (N, Ca, C’(5–12) = 3.00 ± 1.5 Å) and (**b**) representative structure of D-QK in 30% TFE (RMSD (N, Ca, C’(5–12) = 0.46 ± 0.5 Å). The molecules are represented as ribbon.

A tentative structure calculation was conducted with 90 upper distance limits, including 57 intra-residue, 23 short- and 10 medium-range restraints, and 41 angle constraints (Figure 3b). The NMR structural statics are reported in Table S3. The NMR structure of D-QK (Figure 3c) shows that the helical conformation is reduced to a single turn, including residues 9–12. At the same time, the N-terminal region lost its helical conformation in favor of a distorted turn. The comparison of the structures of QK and D-QK (Figure 4a) clearly shows that the insertion of D-amino acids at the N-terminal region of the peptide determines a decrease of the helical populations where only the C-terminal residues preserve a well-defined secondary helical structure.

**Figure 5:**
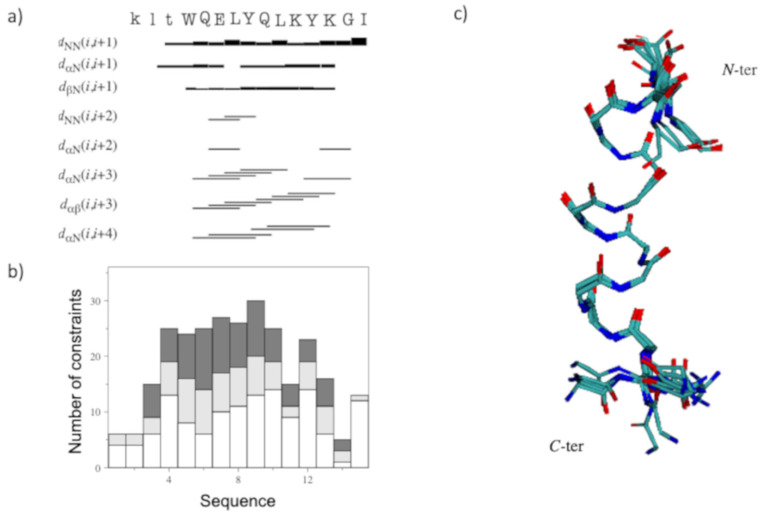
(**a**) D-QK NOEs diagram in H_2_O/TFE 70/30 *v/v*. The amino acids sequence is reported on top. Principal sequential and medium-range NOEs are shown; the thickness of each block is proportional to the corresponding NOE intensity. (**b**) Number of constraints per residue. White, light gray and dark gray bars indicate intra-residue, sequential, and medium-range connectivities, respectively. (**c**) Superposition of the minimized 20 best structures represented as ribbons (RMSD (N, Ca, C’(5–12) = 0.13 ± 0.05 Å). N-ter and C-ter refer to the amino and carboxyl termini, respectively.

Because the addition of TFE leads to a more structured conformation of the peptide according to the CD spectra, the NMR characterization of the D-QK was also performed in 30% TFE. The identification of spin systems and assignment of individual resonances for D-QK together to coupling constants (Table S1 and Table S2) were obtained as previously described for D-QK in aqueous solution. The analysis of the secondary chemical shifts for the Hα protons in 30% TFE points out the presence of helical conformation encompassing the whole peptide sequence with a percentage of helical population equal to 53% (Figure 2), very close to that of QK (59%). The NOESY assignments of the D-QK showed an extensive NN (i, i + 1), αN (i, i + 3), and αβ (i, i + 3) set of cross peaks primarily observed in the 4–12 region of the peptide, as shown in Figure 5a, which confirmed the high helical propensity of D-QK in 30% TFE as already indicated by CD and CSD analyses. The final input for the torsion angle dynamics program (CYANA) for calculation of the structure of D-QK in 30% TFE consisted of a total of 135 NOE constraints (71 intra-residue, 30 short and 34 medium range) and 61 torsion angle restraints (Figure 5b and Table S4). The NMR spectroscopy structure of D-QK (Figure 5c) showed a well-defined helix encompassing residues 4–12 in agreement with the CSI data and very similar to what was observed for the QK peptide (Figure 4b).

### 2.3. Serum Stability Analysis

**Figure 6:**
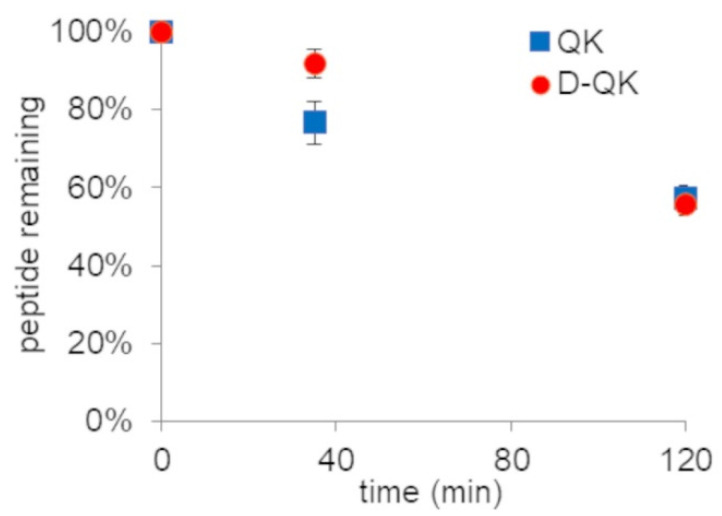
Analysis of peptide stability in 50% human serum. The relative amount of peptide was determined by RP-HPLC measuring the area of the peptide peak revealed at 220 nm. Data are reported as means of a triplicate experiment.

D-amino acids are more resistant to protease degradation. In order to verify the effect of D-amino acid replacement on QK stability in serum, we performed a time-course analysis. Peptides were incubated with 50% diluted human serum and analyzed by RP-HPLC at three different times (0′, 35′ and 120′). The area of the chromatographic peak corresponding to the entire peptide was determined (Figure 6). Overall, peptide QK presents fairly good stability in 50% serum, as almost the 50% is still present after 3 h of incubation. The presence of the D-amino acids at the N-terminal region does not affect the stability of proteases.

### 2.4. Cell Proliferation Analysis

**Figure 7:**
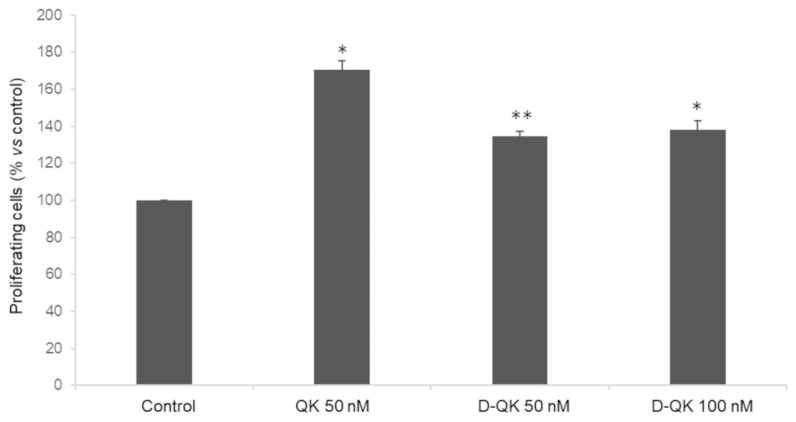
Proliferation assay on HUVEC cells. Cells (1200 cells/well) were treated under starvation conditions for 6 h and incubated with peptides at indicated concentrations for 24 h. Data are expressed as the percentage of proliferating cells with respect to the control (untreated cells) and are expressed as means ± SE of at least three independent experiments (* *p* < 0.05; ** *p* < 0.01).

In order to evaluate the effect of N-Cap chiral inversion on peptide biological activity, we decided to perform a cell proliferation assay using primary human endothelial cells. Peptides were incubated with HUVECs after starvation, and the number of proliferating cells was revealed by fluorescence (Figure 7). As expected, peptide QK at 50 nM induces an increase in the number of cells (71%) with respect to the control. Peptide D-QK, instead, showed a reduced effect with respect to peptide QK. In fact, at the concentrations of 50 and 100 nM, we observed an increase of proliferating cells of 35% and 38%, respectively.

## 3. Discussion

Capping motifs have been identified in proteins as helix start/stop signals [7]. In particular N-capping motifs also contribute to helix stability [35,36]. The design of N-capping motif in short helical peptides, which are inherently unstable in water, has been suggested as a tool to induce the helical conformation. Peptide QK design envisages a capping box sequence (Leu2-Thr3) at its N-terminus based on the statistical positional preference for position N’ and Ncap [7]. Peptide QK folds as stable helix in water starting from Trp4 as expected [9]. However, the NMR structure of the peptide presents frayed peptide ends which prevent the structural determination of the capping motif. Molecular dynamics simulation suggested that the N-terminal region is important for peptide folding and deletion experiments confirmed the role played by the first three QK residues (Lys-Leu-Thr). However, deletion experiments drastically modify the peptide properties. In particular, shortening the sequence by three amino acids in a 15-mer peptide could affect helix stability. We reasoned that changing the chirality of the first three residues will be less impactful on the peptide chemical properties but at the same time allow us to highlight the role played by the N-terminal region. D-amino acids are useful molecular tools to probe conformational aspect in protein and peptides [37,38,39,40,41]. In this work, we replaced the N-terminal region of peptide QK, Lys-Leu-Thr, which precedes the helix start, with the sequence ^D^Lys-^D^Leu-^D^Thr. The QK N-capping motif was modeled on the capping box [42,43]. This motif is characterized by a reciprocal hydrogen bond interaction between the side chain and backbone of N-cap and N residue and a hydrophobic interaction between the side-chain of residue N’ and N3. The mean Φ, Ψ dihedral angle values for N’ and Ncap residue are (−102, +140) and (−86, +150), respectively [7]. Changing the configuration of the N-terminal amino acids should disadvantage the formation of the N-capping motif as the Φ, Ψ values are in the not-allowed region of the Ramachandran plot for a D-residue [44]. 

The QK peptide analogue, namely D-QK, showed a circular dichroism spectrum, suggesting a low folded helical peptide in solution. Hence, measuring the ellipticity at 222 nm, we can estimate a loss of helical content of more than 50% with respect to peptide QK.

In order to analyze the effect of the chiral inversion at the molecular level, we solved the NMR structure of peptide D-QK in water and 30% TFE. Peptide D-QK was characterized by a scarcely populated helical segment with a more defined helical region involving residues from Tyr8 to Tyr12, whereas in peptide QK the helix starts at Trp4 and ends at Tyr12. In both NMR structures, the terminal ends are frayed. Based on the CSD, we estimated for D-QK a helical content of 30%. However, it should be noted that the D-QK peptide is still able to partially assume a helical conformation. Based on the NMR experiments we observed that the main effect of the presence of the all-D N-terminal residues was a distortion of the first helical turn, but the forces driving helix folding are preserved in the remaining part of the peptide. The complete deletion of the N-terminal region has a more dramatic effect on peptide folding, as observed in QK4-15 peptide [15]. It should be considered that D-QK (15 mer) and QK4-15 (12 mer) peptides have different length and QK4-15 peptide might fail to assume a full helical conformation as it could be too short to be sufficiently stable. However, we already reported that QK1-12 peptide (12 mer), sharing with D-QK and QK4-15 peptides the segment 4-12, assumes a well-defined helical conformation [15,16], suggesting that even in a 12-mer peptide the segment 4-12 is able to fold in helical conformation if properly stabilized. D-amino acids as N-capping residues cannot stabilize satisfactorily the first helical turn with respect to an optimized N-capping sequence as in QK peptide. This suggests that the sequence Lys-Leu-Thr in QK effectively plays a role as N-capping motif. However, it is worth nothing that ^D^Lys-^D^Leu-^D^Thr does not fully abolish QK helical content, constraining the full peptide sequence to an unordered structure, but, instead, exerts a local effect, distorting the structure of the first turn.

CD analysis in the presence of TFE demonstrated that the helical content of D-QK peptide increases, as the mean residue ellipticity at 222 shifts from about −1500 (0% TFE) to −8000 (40% TFE), suggesting the formation of a well-defined helical conformation. In order to analyze the structure of the all-D segment and the conformation of the first helical turn, we solved the NMR structure of D-QK in 30% TFE. The NMR analysis confirmed the ability of the peptide to improve its helical organization in 30% TFE. From CSD analysis, we can estimate a 50% helical content for D-QK peptide. In particular, all residues of the segment 4–12 increase their helical content, especially the segment 4–7, suggesting that even in the presence of a stretch of D-residues, i.e., in the absence of N-capping interactions, the first helix turn residues in D-QK have the ability to adopt a predominantly α-helical conformation.

As the introduction of the D-residues affects peptide helical conformation, albeit not completely abolishing its overall ability to fold into a helical structure, which is fundamental for the biological activity of peptide QK, we wanted to verify if D-QK retained the biological activity of peptide QK by performing a cell proliferation assay. Interestingly, peptide D-QK is still able to induce cell proliferation in endothelial cells, but to a lesser extent with respect to peptide QK. Considering that the interacting residues are the same in the two peptides, the different biological activity can be ascribed to the different helical contents of the two peptides. Previously, we already noted that the biological activity of peptide QK depends on the ability to fold as a helical peptide [9,16]. We demonstrated that QK biological activity depends on the ability of the peptide to adopt a helical conformation in solution. Notably, VEGF15, a peptide reproducing the natural VEGF a1 helix, possessing all the residues needed for the interaction with the receptor, but being unstructured in water, has no biological activity [9]. Therefore, we believe that structural preorganization is important for receptor binding, and thus peptide bioactivity. Besides, upon binding to the VEGF receptors exposed on the cell membrane, the D-QK helical conformation could be stabilized, promoting the transition to the functional helical conformation, as we previously observed for another VEGF mimicking peptide [25]. Lastly, we reasoned that the D-amino acid should confer to peptide QK an improved biological stability in vivo which might compensate for the reduced biological activity. The analysis of D-QK stability in human serum showed that the stability of the proteases is unaffected, suggesting that the main proteases cleavage sites are located elsewhere in the sequence.

## 4. Materials and Methods

### 4.1. Peptide Synthesis

D-QK peptide (*Ac*-^d^Lys-^d^Leu-^d^Thr-Trp-Gln-Glu-Leu-Tyr-Gln-Leu-Lys-Tyr-Lys-Gly-Ile-*NH_2_*) was synthesized on solid phase by standard Fmoc chemistry. The synthesis was performed on a Rink Amide-ChemMatrix^®^ resin (Biotage, Uppsala, Sweden) (loading 0.52 mmol g^−1^) at 0.1 mmol scale. Each amino acid coupling reaction was performed for 30 min, using 5 eq of the Fmoc-protected amino acid (Iris Biotech, Marktredwitz, Germany), 4.9 eq of the activating agent 1-[Bis(dimethylamino)methylene]-1*H*-1,2,3-triazolo [4,5-b]pyridinium 3-oxid hexafluorophosphate (HATU) (Iris Biotech), and 10 eq of the base *N,N*-diisopropylethylamine (DIPEA) (Sigma-Aldrich, Milan, Italy), in dimethylformamide (DMF) (Carlo Erba, Milan, Italy). After each amino acid coupling, a capping reaction (5 min, under stirring) was performed by treating the resin with a solution of 2 M acetic anhydride (Sigma-Aldrich), 0.55 M DIPEA in *N*-methyl-2-pirrolidone (NMP) (Romil, Cambridge, UK). This capping solution was also used to perform the N-terminal acetylation. Fmoc removal was performed using a solution of with 20% piperidine (Sigma-Aldrich) in DMF, twice for 5 min. After each reaction step, the resin was washed with DMF. All the reaction steps were performed under stirring and at room temperature. Final cleavage and deprotection was performed by incubating the resin with a solution of trifluoracetic acid (TFA) (Sigma-Aldrich) /H_2_O/triisopropylsilane (Sigma-Aldrich) (95:2.5:2.5, *v/v/v*) for 3 h at room temperature. The resin was then filtered, and the crude peptide was precipitated in ice-cold diethyl ether (Sigma-Aldrich) and harvested by centrifugation. The crude peptide pellet was washed twice with diethyl ether, then resuspended in 5 mL of H_2_O/CH_3_CN 90/10 and lyophilized. The D-QK peptide was finally purified by RP-HPLC on a preparative HP 1200 Series system (Agilent Technologies, Santa Clara, CA, USA) using a column Axia 50 × 21.2 mm, Synergi, 4 µm, Fusion RP 80 Å (Phenomenex, Torrance, CA, USA) and applying a gradient of CH_3_CN (0.1% TFA) in H_2_O (0.1% TFA) from 20% to 50% in 20 min (20 mL min^−1^). D-QK peak fractions were pooled and lyophilized. Hence, 13.6 mg of pure peptide were obtained. D-QK purity was verified by analytical RP-HPLC, resulting >95% based on the chromatographic peak area revealed at 210 nm. The analysis was performed on an analytical HP 1200 Series system (Agilent Technologies, Santa Clara, CA, USA), equipped with a column Jupiter Proteo 150 × 4.6 mm, 90 Å, 4 µm and applying a gradient of gradient of CH_3_CN (0.1% TFA) in H_2_O (0.1% TFA) from 20% to 40% in 20 min (1 mL min^−1^) (retention time 15.99 min). D-QK peptide identity was confirmed by mass analysis on an Agilent LC-MS 1200 Infinity Series (Agilent Technologies) equipped with a diode array and ESI-ToF detectors, fitted with a column Jupiter C18 150 × 2 mm, 300 Å, 3 µm (Phenomenex) and applying a gradient of CH_3_CN (0.05% TFA) in H_2_O (0.05% TFA) from 5% to 70% in 20 min (0.2 mL min^−1^) (monoisotopic MW_calc_: 1951.082 Da; monoisotopic MW_exp_: 1951.092 Da).

### 4.2. Circular Dichroism Spectroscopy

Circular dichroism (CD) spectroscopy studies on the D-QK peptide were performed using a Jasco J-715 spectropolarimeter equipped with a PTC-423 Peltier temperature controller. CD spectra were recorded as average of three scans, at 20 °C, in the far-UV wavelength range 260–190 nm, using a scanning speed of 10 nm min^−1^, a band width of 1 nm, a data pitch of 0.1 nm, a response of 2 sec, and using a 0.1 cm path-length quartz cuvette (Hellma). CD spectra were recorded at 100 µM peptide concentration in filtered and degassed 5 mM potassium phosphate buffer, pH 7.0, and in filtered and degassed H_2_O containing an increasing concentration of the cosolvent trifluoroethanol (TFE) (Sigma-Aldrich) (0, 5%, 10%, 15%, 20%, 30%, 40% of TFE). Peptide concentration was determined by UV-spectroscopy, measuring the absorbance at 280 nm of the peptide solution and using the Lambert-Beer equation and the molar extinction coefficient of 8480 M^−1^ cm^−1^. All CD spectra have been smoothed and the buffer or solvent contribution has been subtracted using the Spectra Manager software. CD data are expressed as mean residue ellipticity [θ].

### 4.3. Nuclear Magnetic Resonance Spectroscopy

NMR experiments were recorded at room temperature with a Varian Unity 600 MHz spectrometer equipped with a cold probe. 1D [1H] and 2D [1H-1H] spectra were acquired for D-QK peptide dissolved in H_2_O/D_2_O 90/10 *v/v*, pH 5.5 and in H2O /TFE_d3_ 70/30 *v/v*, pH 5.5, at peptide concentrations equal to 0.5 mM. The sample was placed in 5 mm NMR tube containing 4 4-dimethyl-4-silapentane-1-sulfonic acid (DSS) as the internal reference for 1H chemical shifts. NMR analyses were carried out by using 2D [1H-1H] TOCSY [45], NOESY [46], and DQF-COSY [47] spectra. Hence, 2D NMR experiments were normally recorded with 16–64 scans, 128–256 FIDs in t1, 1024 or 2048 data points in t2. TOCSY and NOESY spectra were obtained by using 70 and 250 ms mixing times respectively. Water suppression was achieved by excitation sculpting [48].

Proton resonance assignment for D-QK was performed by following the well-established sequential specific methodology based on homo-nuclear spectra [49]. Hence, the 1D 1H spectrum was analyzed by using ChemAxon software (http://www.chemaxon.com, accessed on 28 September 2022) while two-dimensional spectra were processed with SPARKY [50] and analyzed with NEASY [51] contained in CARA (http://cara.nmr.ch, accessed on 28 September 2022). Chemical shift deviations (CSDs) of Hα protons from random coil vales were estimated with the protocol suggested by Kjaergaard and collaborators [52].

To estimate the helix population, the observed Hα chemical shift values were compared with the random coil values (ΔδHα = δHα_observed_ − δHα_random coil_). Then, the average ΔδHα values for all helical residues were divided by the ΔδHα value, corresponding to 100% helix (−0.39 ppm for the Hα [53] and multiplied by 100% [54].

Peptide structure calculations were performed with CYANA 2.1 program [55]. Distance constraints were determined from 2D [1H-1H] NOESY spectra and angular constraints were generated with the GRIDSEARCH module of CYANA. Structure calculations were initiated from 100 random conformers. The 20 structures with the lowest CYANA target functions were extensively examined with the program MolMol [56]. Figures with 3D structures were generated either with MolMol or Pymol [57].

### 4.4. Serum Stability Analysis

Briefly, 20 µL of peptide (10 mg/mL stock solution in H_2_O) were added to 200 µL of 50% aqueous solution of human serum (Sigma-Aldrich) (previously centrifuged at 10,000 rpm for 10 min at 4 °C) and the mixture was incubated at 37 °C. At each time point (0, 30, 120 min), an aliquot of 6 µL was diluted in a ten times volume of H_2_O and analyzed by RP-HPLC on an Atlantis C18-column (Waters, 5 µm, 4.6 mm × 150 mm) using a linear aqueous acetonitrile gradient containing TFA (0.1%, *v/v*). Initial condition 5% B, 5−5% B in 5 min, 5−40% B in 27 min, 40−100% B in 5 min, and flow rate 1 mL/min (B = acetonitrile 0.1% TFA). Peptides were detected by recording the absorbance at 220 nm and quantified by their peak areas relative to the initial peak areas (0 min). All stability tests were performed at least in triplicates.

### 4.5. Cell Culture and Conditions

Human umbilical vein endothelial cells (HUVEC) were purchased from Lonza (Basel, Switzerland). All experiments were performed using low passage cell cultures. Cells were grown in EGM-2 (endothelial cell growth medium-EBM2 supplemented by BulletKit) from Lonza. Cells were maintained in humidified air containing 5% CO_2_, at 37 °C.

### 4.6. Cell Proliferation Assay

HUVEC were seeded at density of 1200 cells/well in 96-well microplates (Becton Dickinson Bioscience). After 6 h of incubation in starvation medium (EBM-2, heparin 0.1%, BSA 0.1%, 1% glutamine), cells were treated with 50 nM or 100 nM peptides. Cell proliferation was determined using the CyQUANT NF Cell Proliferation Assay Kit (Invitrogen, Monza, Italy) after 24 h treatment. In detail, the medium was removed, and the cells were incubated with CyQUANT NF reagent for 1 h at 37 °C according to the manufacturer’s instructions [26]. Subsequently, the plates were analyzed using a microplate reader (Hamilton, Reno, USA) with an excitation wavelength of 485 nm and an emission wavelength of 520 nm. The results are presented as the percentage of proliferating cells versus the control (untreated cells) and are expressed as means ± SD of at least three independent experiments. Statistical significance was determined by a paired, two-sided Student’s test, where a *p* value less than 0.05 was significant.

## 5. Conclusions

The N-capping sequence is important to stabilize the helical conformation in the water of QK, a short-medium size peptide. The presence of D-amino acids at the N-terminal of a peptide sequence able to fold in a helical structure decreases the overall peptide helical content, mainly distorting the conformation of the first helical turn residues. 

## Data Availability

Not applicable.

## Supplementary Materials

The following supporting information can be downloaded at: https://www.mdpi.com/article/10.3390/molecules27206982/s1. Figure S1: RP-HPLC chromatographic and ESI-mass spectrum of pure D-QK; Table S1: Chemical shifts of D-QK protons in water and in water/TFE 70/30 *v/v*; Table S2: Coupling constants of D-QK protons in water and in water/TFE 70/30 *v/v*; Table S3: NMR structural statistics of the D-QK peptide in H2O; Table S4: NMR structural statistics of the D-QK peptide in H_2_O/TFE 70/30.
