# Supplementary material for: Switching the N-Capping Region from all-L to all-D Amino Acids in a VEGF Mimetic Helical Peptide
Source: Molecules. 2022 Oct 17;27(20):6982. doi: 10.3390/molecules27206982 (PMC9607104; doi:10.3390/molecules27206982)
Supplement: Supplementary file 1 [file molecules-27-06982-s001.zip › molecules-1970830-supplementary.pdf]

molecules

MDPI

Supplementary Materials

Switching the N-Capping Region from all-L to all-D Amino Acids in a VEGF Mimetic Helical Peptide

List of contents:

- Figure S1. RP-HPLC chromatographic and ESI-mass spectrum of pure D-QK.
- Table S1. Chemical shifts of D-QK protons in water and in water/TFE 70/30 v/v.
- Table S2. Coupling constants of D-QK protons in water and in water/TFE 70/30 v/v.
- Table S3. NMR structural statistics of the D-QK peptide in H2O
- Table S4. NMR structural statistics of the D-QK peptide in H2O/TFE 70/30.

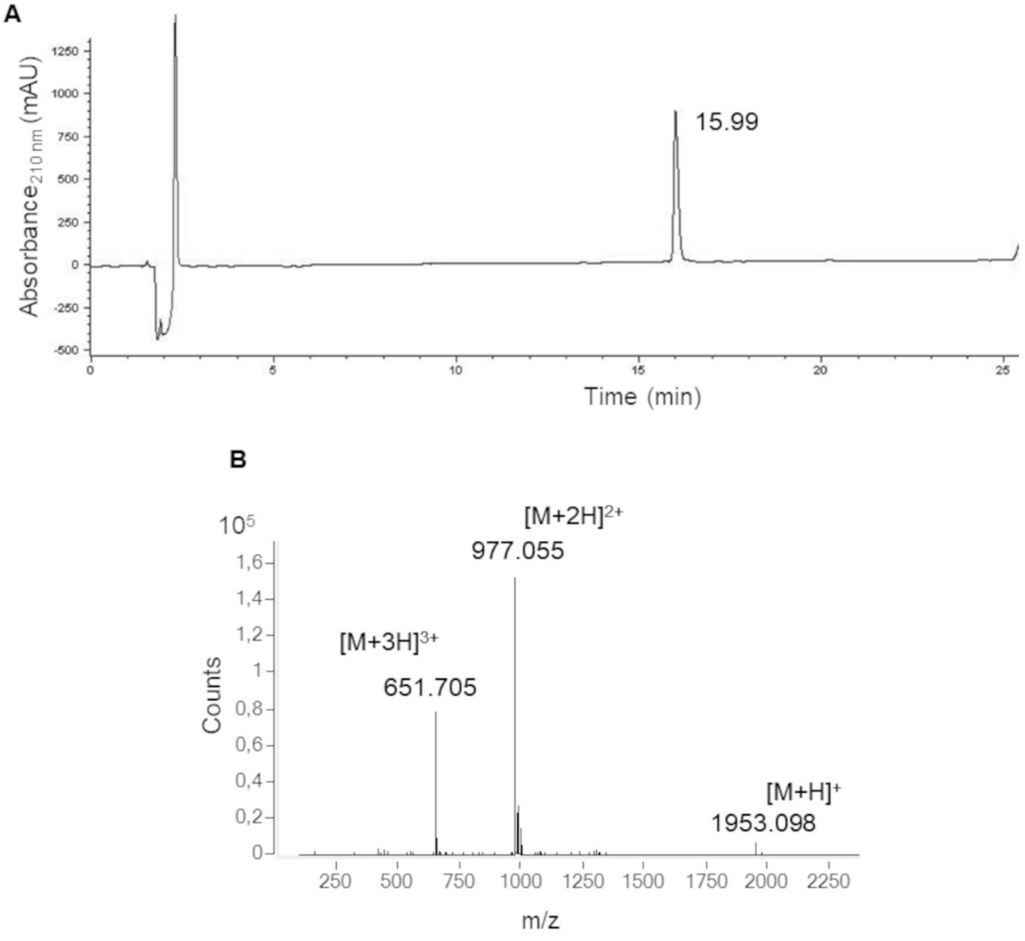

Figure S1. (**A**) RP-HPLC chromatographic profile revealed at 210 nm of pure D-QK. (**B**) ESI-mass spectrum of the species eluted at 15.99 min.

Table S1. Chemical shifts of D-QK protons in water and in water/TFE 70/30 v/v.

Residue	Proton	Chemical Shift, ppm (H_2O)	Chemical Shift, ppm (H_2O/TFE 70/30 v/v)
**D-Lys1**	HN	8.08	7.86
	Hα	4.31	4.49
	Hβ2-Hβ3	-	1.78
**D-Leu2**	HN	8.30	8.21
	Hα	4.13	4.24
	Hβ2-Hβ3	1.49	1.65
	Hγ	1.35	1.51
**D-Thr3**	HN	7.96	8.19
	Hα	4.47	4.36
	Hβ	4.58	4.48
	Hγ21-Hγ22-Hγ23	1.37	1.16
**Trp4**	HN	8.07	7.84
	Hα	4.60	4.46
	Hβ2-Hβ3	3.21	3.33
	Hδ1	7.21	7.29
	Hε1	10.1	9.98
	Hζ3	7.15	7.10
	Hζ2	7.46	7.44
	Hη2	7.25	7.14
**Gln5**	HN	8.23	8.56
	Hα	4.05	3.85
	Hβ2-Hβ3	2.00	1.89
	Hγ2-Hγ3	2.11	2.18
	Hε21	6.82	6.78
	Hε22	7.40	7.35
**Glu6**	HN	8.11	7.81
	Hα	4.21	4.09
	Hβ2-Hβ3	1.93/1.84	2.07/1.95
	Hγ2-Hγ3	2.27	2.36
**Leu7**	HN	8.09	7.72
	Hα	4.21	3.95
	Hβ2-Hβ3	1.67/1.38	1.82/1.70
	Hγ	1.51	1.52
	Hδ11- Hδ12- Hδ13	0.90	0.99
	Hδ21- Hδ22- Hδ23	0.80	0.89
**Tyr8**	HN	7.96	8.40
	Hα	4.39	4.17
	Hβ2-Hβ3	3.00/2.85	3.11/3.05
	Hδ1- Hδ2	7.08	7.15
	Hε1– Hε2	6.81	6.79
**Gln9**	HN	8.02	7.92
	Hα	4.17	4.09
	Hβ2-Hβ3	1.99/1.92	2.24/2.19
	Hγ2-Hγ3	2.24	2.56/2.47
	Hε21	6.70	6.74
	Hε22	7.33	7.30
**Leu10**	HN	8.98	7.69
	Hα	4.16	4.15
	Hβ2-Hβ3	1.58/1.48	1.88/1.80
	Hγ	1.20	1.61
	Hδ11- Hδ12- Hδ13	0.85	0.89

	Hδ21- Hδ22- Hδ23	0.77	-
**Lys11**	HN	7.99	8.14
	Hα	4.19	4.17
	Hβ2-Hβ3	1.10	1.18/1.15
	Hγ2-Hγ3	1.53	1.58
**Tyr12**	HN	8.06	8.04
	Hα	4.48	4.43
	Hβ2-Hβ3	2.97/2.83	3.08/2.76
	Hδ1- Hδ2	7.06	7.08
	Hε1– Hε2	6.77	6.80
**Lys13**	HN	8.23	7.90
	Hα	4.28	4.23
	Hβ2-Hβ3	1.87	1.96
	Hγ2-Hγ3	1.78	1.71
**Gly14**	HN	7.84	7.99
	Hα	3.94	3.96
**Ile15**	HN	7.91	8.07
	Hα	4.11	4.06
	Hβ	1.76	1.70
	Hγ	0.95	1.00
	Hγ12– Hγ13	1.45	1.22

The proton chemical shifts of D-QK are relative to water protons (4.75 ppm).

Table S2. Coupling constants of D-QK protons in water and in water/TFE 70/30 v/v.

Residue	Coupling Constants, Hz (H_2O)	Coupling Constants, Hz (H_2O /TFE 70/30 v/v)
Lys1	-	-
Leu2	-	7.5
Thr3	7.0	6.5
Trp4	6.8	6.5
Gln5	6.1	6.0
Glu6	6.0	6.1
Leu7	-	6.2
Tyr8	7.0	6.0
Gln9	6.5	6.2
Leu10	6.5	6.0
Lys11	-	-
Tyr12	7.0	6.5
Lys13	7.0	7.0
Gly14	-	-
Ile15	8.0	7.8

Table S3. NMR structural statistics of the D-QK peptide in H_2O.

PARAMETER	VALUE
**NOE upper distance limit**	90
Intra-residue	57
Short distance	23
Medium/long distance	10
**Number of dihedral angle constraints**	41
**Residual target function, Å**	0.43 ± 0.05

**Residual NOE violations**	
Number >0.1	±1
Maximum, Å	0.18 ± 0.03
**Residual angle violations**	
Number >2.0	0 ± 0
Maximum, Å	0
**R.M.S.D.[a] to the mean coordinates, Å**	
N–Cα–C′ (5–12)	0.37 ± 0.05
All heavy atoms (5–12)	1.17 ± 0.11
**Ramachandran plot residues[b] (%)**	
In most favored regions	60.0
In additional allowed regions	33.9
In generously allowed regions	6.1
In disallowed regions	0.0
a Calculated by MOLMOL. b Calculated by CYANA 2.1.	

Table S4. NMR structural statistics of the D-QK peptide in H_2O/TFE 70/30.

PARAMETER	VALUE
**NOE upper distance limit**	135
Intra-residue	71
Short distance	30
Medium/long distance	34
**Number of dihedral angle constraints**	61
**Residual target function, Å**	0.38 ± 0.05
**Residual NOE violations**	
Number >0.1	±1
Maximum, Å	0.15 ± 0.02
**Residual angle violations**	
Number >2.0	0 ± 0
Maximum, Å	0
**R.M.S.D.[a] to the mean coordinates, Å**	
N–Cα–C′ (5–12)	0.13 ± 0.05
All heavy atoms (5–12)	0.99 ± 0.06
**Ramachandran plot residues[b] (%)**	
In most favored regions	88.3

In additional allowed regions	11.7
In generously allowed regions	0.0
In disallowed regions	0.0
a Calculated by MOLMOL. b Calculated by CYANA 2.1.	
